# Immortality, but not oncogenic transformation, of primary human cells leads to epigenetic reprogramming of DNA methylation and gene expression

**DOI:** 10.1093/nar/gkt1351

**Published:** 2013-12-26

**Authors:** Katrina Gordon, Thomas Clouaire, Xun X. Bao, Sadie E. Kemp, Maria Xenophontos, Jose Ignacio de Las Heras, Irina Stancheva

**Affiliations:** Wellcome Trust Centre for Cell Biology, School of Biological Sciences, University of Edinburgh, Michael Swann Building, Mayfield Road, Edinburgh EH9 3JR, UK

## Abstract

Tumourigenic transformation of normal cells into cancer typically involves several steps resulting in acquisition of unlimited growth potential, evasion of apoptosis and non-responsiveness to growth inhibitory signals. Both genetic and epigenetic changes can contribute to cancer development and progression. Given the vast genetic heterogeneity of human cancers and difficulty to monitor cancer-initiating events *in vivo*, the precise relationship between acquisition of genetic mutations and the temporal progression of epigenetic alterations in transformed cells is largely unclear. Here, we use an *in vitro* model system to investigate the contribution of cellular immortality and oncogenic transformation of primary human cells to epigenetic reprogramming of DNA methylation and gene expression. Our data demonstrate that extension of replicative life span of the cells is sufficient to induce accumulation of DNA methylation at gene promoters and large-scale changes in gene expression in a time-dependent manner. In contrast, continuous expression of cooperating oncogenes in immortalized cells, although essential for anchorage-independent growth and evasion of apoptosis, does not affect *de novo* DNA methylation at promoters and induces subtle expression changes. Taken together, these observations imply that cellular immortality promotes epigenetic adaptation to highly proliferative state, whereas transforming oncogenes confer additional properties to transformed human cells.

## INTRODUCTION

It is widely recognized that tumours and tumour-derived cell lines exhibit altered patterns of DNA methylation and gene expression in comparison with normal tissues and primary cells. Gain of DNA methylation at normally DNA methylation-free gene promoters and extensive loss of DNA methylation throughout the genome have been detected in a variety of tumour types (1–4). Aberrant methylation of gene promoters can lead to stable silencing of tumour suppressor genes and constitutes an alternative mechanism to genetic loss of gene function that can be brought about by mutations, deletions and chromosomal rearrangements (1,3,4). Loss of DNA methylation from repetitive sequences is thought to promote genomic instability, which often accompanies cancer progression (5,6).

Despite the wealth of data documenting these findings, it is largely unclear when and how the changes in DNA methylation occur in transformed human cells (3). Tumours usually initiate from a small number of mutant cells, and these tumour-initiating cells are difficult to detect, isolate and monitor in long-term studies (7). Similar limitations apply to most available mouse cancer models. The vast majority of epigenetic studies on human cancers are carried out either on limited amount of clinical material isolated from patients when the disease is well advanced or on cell lines established from tumours and maintained in culture for extended periods of time. Although data indicating strong correlation between accumulated epimutations and tumour grade/type are available for colon, lung, prostate and breast cancer (8–11), the precise timing of the initial methylation events and the progression of epigenetic alterations in human cells undergoing tumourogenic transformation have been difficult to estimate due to the vast genetic heterogeneity of human cancers. In most cases, it is extremely challenging to determine the precise relationship between genetic background, oncogenic mutations, genomic instability and detected epigenetic changes (12).

To circumvent these limitations and generate a cancer model system amenable to long-term tracking of epigenetic events and further mechanistic studies, we used an established method to transform human somatic cells *in vitro* using a combination of well-defined factors (13). We established isogenic immortalized and transformed human cell lines derived from primary foetal lung fibroblasts (MRC-5) and followed the temporal changes in gene expression and DNA methylation at gene promoters in these independent, but related to each other, cell populations. Our analyses show that MRC-5 cells, immortalized by expression of human telomerase reverse transcriptase (hTERT) catalytic subunit, and transformed MRC-5 cells, expressing hTERT, SV40 large T-antigen (T-Ag) and constitutively active oncogenic H-RAS^GV12^, progressively accumulate extensive changes in gene expression and *de novo* DNA methylation at gene promoters that become apparent after 50 population doublings (pd) in culture. Remarkably, *de novo* DNA methylation at gene promoters occurred at specific loci with similar timing in both the immortalized and transformed cell lines suggesting that gain of DNA methylation does not require expression of oncogenes. The accumulation of DNA methylation at gene promoters took place predominantly at genes that were transcriptionally inactive in the parental cell line, but did not correlate with pre-existing Polycomb-dependent H3K27 trimethylation (H3K27me3) previously reported to pre-mark promoters for *de novo* DNA methylation (14–16). Importantly, immortalized and transformed cell lines displayed different gene expression profiles, indicating that the presence of oncogenes modulates the properties of immortal cells. Our data demonstrate that programmed *de novo* DNA methylation at specific loci and adaptation of transcriptional output of the genome to a highly proliferative state can occur in diploid human cells without a major input from oncogenic proteins. On the other hand, transforming oncogenes contribute to further modulation of gene expression and promote evasion of apoptosis and anchorage-independent growth, which are essential properties of cancer cells.

## MATERIALS AND METHODS

### Cell lines and viral infections

The human male foetal lung fibroblast cell line MRC-5 (ATCC number: CRL-171) and all MRC-5-derived cells were cultured in MEM (Life Sciences) supplemented with 10% foetal calf serum, 1 mM non-essential amino acids, 1 mM sodium pyruvate, 100 U/ml penicillin, 1 mg/ml of a streptomycin and 2 mM l-glutamine. The pBABE-Neo-hTERT, pBABE-Hygro-SV40 T-Ag and pBABE-Puro-H-RAS^V12G^ plasmids were packaged into retroviral particles in amphotropic Phoenix A cell line. Culture supernatants were harvested 48 h later and the retroviral titres determined by infection of NIH-3T3 mouse fibroblast cells. The MRC-5^hTERT^ cell line was generated by infecting 10^5^ MRC-5 cells with pBABE-Neo-hTERT retroviral particles at multiplicity of infection (MOI) = 1 in the presence of 4 µg/ml polybrene. After a 7-day selection with 250 µg/ml G418, drug-resistant colonies were pooled and designated as passage 1. The MRC-5^TSR^ cell line was generated by infecting the MRC-5^hTERT^ with retroviral particles carrying pBABE-Hygro-T-Ag and subsequently after selection with 150 µg/ml hygromycin for 7 days with packaged pBABE-Puro-H-RAS^V12G^. Selection with 1 µg/ml puromycin was applied and resistant cells were pooled and designated as passage 1.

### Growth curves

A total of 5×10^4^ cells were initially seeded (cell input = ***n***_0_) into six-well dishes, and the cell yields (***n***) were recorded at each passage and the population doublings calculated from the formula MPD (mean population doubling) = 3.32 (log_10_ ***n***- log_10_ ***n***_0_).

### Soft agar assays

A bottom layer of 10 ml 1.6% agar (BioGene) in MRC-5 growth medium was prepared in 10-cm tissue culture dishes and allowed to solidify. Each cell line (set up in triplicate) was seeded at a density of 10^5^ cells/dish in a 4 ml top layer of 0.8% agar in MRC-5 growth medium. Cells were incubated at 37°C with weekly overlays with 4 ml of top layer agar without cells. After 6–8 weeks colonies were scored blind.

### Telomere repeat amplification protocol

Cell extracts were prepared and assayed for telomerase activity using the TRAPeze kit (Millipore), following the manufacturer’s instructions. Pilot experiments were initially carried out on a range of protein concentrations (0.2–2 µg) to determine the linear range for each cell line. Assays were routinely carried out using 1 µg of protein extract for each cell line. The polymerase chain reaction (PCR) products were resolved on 10% non-denaturing polyacrylamide gel, visualized by staining with SYBR Green and scanned at 473 nm on FLA-5100 scanner.

### Telomere restriction fragment Southern blots

Five microgram of genomic DNA prepared from each of the cell lines was digested with Hinf I and Rsa I restriction enzymes overnight at 37°C. The digests were resolved in a 1% Tris–Acetate–EDTA gel and transferred to Zeta-Probe GT membrane (BioRad) with 0.4 M NaOH for 16 h. The DNA was cross-linked to the membrane using a ultraviolet cross-linker (Stratagene) set on autocross-link mode (120 000 µJoules) and the blot hybridized with a radiolabelled oligonucleotide probe [(TTAGGG)_3_] at 42°C overnight in buffer containing 1 mM ethylenediaminetetraacetic acid (EDTA), 0.5 M NaHPO4 and 7% sodium dodecyl sulphate (SDS). The blot was subsequently washed with 3 × saline-sodium citrate (SSC), 0.1% SDS at 42°C and exposed to X-ray film at −70°C. 

### Western blots

Total cell extracts were prepared from 1×10^7^ cells by resuspending the cell pellet in 2 ml of lysis buffer (10 mM Tris–HCl pH 7.5, 1 mM MgCl_2_, 1 mM EGTA, 5 mM β-mercaptoethanol, 0.5% Cholamidopropyl- dimethylammoniopropanesulfonate (CHAPS) (Sigma C-5849) and 10% glycerol) and incubated on ice for 30 min. The cells were centrifuged at 13 000 rpm for 30 min at 4°C and supernatants taken. Nuclear extracts were prepared as previously described (17). Fifty microgram of either total cell protein extract or nuclear extract prepared from indicated cell lines was resolved on either 10 or 15% sodium dodecyl sulphate–polyacrylamide gel electrophoresis gels and transferred to polyvinylidene fluoride (PVDF) membrane (Biorad). The blots were probed with anti-SV40 T-antigen (Santa Cruz sc-148), anti- H-RAS (Santa Cruz sc-520), anti HDAC-1 (Santa Cruz sc-7872) and anti-tubulin (Cancer Research UK) antibodies in 1×Tris-buffered saline (TBS) buffer with 0.1% Tween 20 followed by appropriate secondary anti-mouse IR800 and anti-rabbit IR670 antibodies (LiCOR Biosciences). Images were collected on Odyssey scanner (LiCOR Biosciences) and quantified with Image Studio software (LiCOR Biosciences).

### Immunohistochemistry

A total of 2 × 10^5^ cells were seeded onto 19-mm coverslips in six-well dishes and 0.6 µM H_2_0_2_ added for 2 h. Cells were then washed with phosphate-buffered saline (PBS) and allowed to recover overnight in growth media. After fixation in 3% formaldehyde for 15 min, the cells were incubated in blocking solution (PBS with 5% bovine serum albumin and 0.3% Triton X-100) for 1 h and then incubated with anti-p21 antibody (Cell Signalling # 2947) overnight at 4°C. The cells were rinsed three times with PBS and incubated for 2 h with Alexa 488 conjugated secondary antibody. Finally, the cells were washed with PBS, counter stained with diamidino-2-phenylidole (DAPI) and mounted in Prolong Gold (Life Sciences). Images were taken at 20× magnification on Olympus BX61 fluorescence microscope equipped with ColorViewII camera and AnalySIS software.

### Methylated DNA affinity purification

Affinity purification of methylated DNA (MAP) was carried out essentially as described (18). Briefly, genomic DNA from MRC-5 cells, MRC-5^hTERT^ (50 and 100 pd) and MRC-5^TSR^ (50 and 100 pd) was digested with MseI, and 50 µg of digested DNA was loaded onto 1 ml chromatography column (Tricorn, GE Healthcare) containing 50 mg of His-tagged methyl-CpG binding domain of MeCP2 protein bound to nickel-charged Chelating Sepharose Fast Flow (GE Healthcare). The column was washed with 10 volumes of buffer A [20 mM HEPES pH 7.5, 100 mM NaCl, 0.1% Tween 20, 10% glycerol, 0.5 mM phenylmethylsulfonyl fluoride (PMSF)] followed by buffer A with increasing concentration of NaCl (0.1–0.7 M). Methylated DNA was eluded with buffer A containing 1 M NaCl. Triplicate runs were done for each of the cell lines and methylated fractions identified by PCR for known methylated regions. The methylated fractions were pooled, concentrated and subjected to whole-genome amplification (WGA kit, Sigma Aldrich) alongside MseI-digested input genomic DNA. Amplified samples were labelled with Cy3 and Cy5, respectively, and co-hybridized to H18-RefSeq promoter microarrays (Roche NimbleGen). The data from promoter microarray experiments can be assessed at ArrayExpress, accession number E-MTAB-2005.

### Promoter microarray data analyses

The normalization of the microarray data and analyses of promoter regions were carried out with custom-designed software ‘Prometheus’ essentially as described previously (17). Briefly, raw fluorescent intensity values were Loess normalized using LIMMA package in R, and the log_2_ values for either MAP/input or ChIP/input were calculated for each individual probe. Subsequently, for comparison between microarray experiments, the probe values were scaled to have the same median absolute deviation. The log_2_ values of all probes located within a 1000-bp window around the transcription start site (TSS) (+500 to −500) were aggregated into a single median log_2_ value for each promoter.

### Chromatin immunoprecipitation

Chromatin immunoprecipitations (ChIPs) were carried out in triplicate as described (19) with antibodies against acetylated H3 (Millipore, 06–599) and H3K27me3 (Millipore, 07–449). ChIP and input DNA were amplified using WGA kit (Sigma), labelled with Cy-dyes and hybridized to H18-RefSeq promoter microarrays (Roche NimbleGen). Microarray data were analysed as described above. The data from ChIP experiments can be assessed at ArrayExpress, accession number E-MTAB-2004.

### Sodium bisulphite DNA sequencing

Sodium bisulphite treatment was carried out essentially as described (20) and processed for sequencing as outlined in (21). PCR primers were designed manually or using MethPrimer software (22) and are available on request. The PCR products were cloned using the CloneJet PCR Cloning Kit (Thermo Scientific) and sequenced using BigDye Terminator v3.1 reagents (Applied Biosystems). BiQ Analyzer software (23) was used to analyse the methylation status of sequences.

### Methylated DNA immunoprecipitation

Methylated DNA immunoprecipitation (MeDIP) was performed as described (24) with minor modifications. Genomic DNA was fragmented by sonication to 300–1000 bp. For each immunoprecipitation, 4 µg of denatured sonicated DNA was incubated for 2 h at 4°C with 10 µl of anti-5-methylcytosine monoclonal antibody (Eurogenetec). A total of 40 µl of M280 Dynabeads conjugated with sheep anti-mouse IgG (Dynal Biotech) were added and incubated for a further 2 h before washing with IP buffer (10 mM Na_2_HPO_4 _pH 7.0; 140 mM NaCl; 0.05% Triton X-100). Proteinase K digestion was carried out at 50°C overnight and the methylated DNA was recovered using Pure-Link PCR Purification Kit (Life Sciences).

### Quantitative PCR

Quantitative PCR was carried out with SYBR Green Master Mix (Roche) according to manufacturer’s instructions on a LightCycler 480 (Roche). Quantitative PCR on MeDIP samples was carried out using 2 μl of MeDIP DNA and 20 ng of total input DNA. Enrichments in the MeDIP fraction were calculated as a percentage of input. Quantitative reverse transcription PCRs were carried in triplicate using independent complementary DNA (cDNA) synthesis reactions (Superscript II; Life Sciences) as template. Three independent RNAs preparations from three different flasks of cells were used for cDNA synthesis. Fold changes relative to Glyceraldehyde-3-phosphate dehydrogenease (GAPDH) were calculated using the Pfaffl method (25). Primer sequences are available on request. 

### Gene expression analyses

Total RNA was purified using Trizol reagent (Life Sciences), and double-stranded cDNA was synthesized from 10 µg of total RNA using SuperScript double-stranded cDNA synthesis kit (Life Sciences) according to manufacturer’s instructions. All samples were labelled with Cy3 dye and hybridized in triplicate to Human Gene Expression H18 Build 4×75 K expression microarrays (Roche NimbleGen). Raw intensity values were quantile normalized using the BioConductor package LIMMA. The log_2_ values of the probes associated with each transcript were summarized into a single log_2_ value using median polish procedure. A linear model was fit to the data with LIMMA, calculating the expression ratio M = log_2_ and moderated *t*-statistics, adjusting *P*-values for multiple testing. The false discovery rate was obtained using the Benjamini–Hochberg method and false discovery rate <0.05 cut-off applied to all M values. The raw data from expression microarray experiments can be downloaded from ArrayExpress, accession number E-MTAB-2003. Functional gene annotation was performed by DAVID (http://david.abcc.ncifcrf.gov) (26).

### Analyses of IMR90 histone modifications data

Histone modification data for IMR90 cell line corresponding to the set of methylated promoters in MRC-5^hTERT^ cells at 100 pd were extracted from Ensembl API (version 67) using HMoTF package of script written in Perl, which retrieved ChIP-seq data from annotated peaks taking into account the genomic coordinates of the analysed regions. The data were converted to *Z*-scores and plotted as heat maps using the open source TIGR MultiExperiment Viewer (MeV) software.

## RESULTS

### Generation of immortalized and transformed cell lines

**Figure 1.**
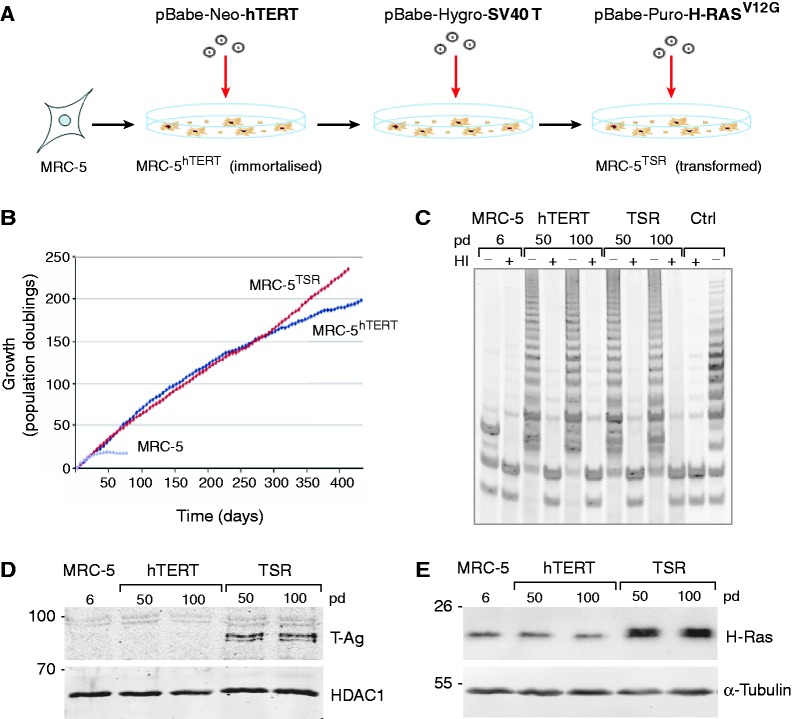
Generation of immortal and transformed human cell lines. (**A**) Immortalized (MRC-5^hTERT^) and transformed (MRC-5^TSR^) human cell lines were generated from embryonic lung fibroblasts MRC-5 by stepwise infection with retroviral particles driving the expression of hTERT, SV40 T-Ag and H-RAS^V12G^. ‘Neo’, ‘Hygro’ and ‘Puro’ indicate drug resistance markers neomycin, hygromycin and puromycin, respectively, carried by the retroviral vectors. (**B**) Growth of MRC-5, MRC-5^hTERT^ and MRC-5^TSR^ cell lines measured as population doublings over 400 days in culture. The parental MRC-5 cell line enters senescence after ∼20 pd. (**C**) Telomerase activity detected by TRAP in cell extracts from MRC-5, MRC-5^hTERT^ and MRC-5^TSR^ cells. ‘Ctrl’ is a control telomerase-positive NCI-H520 lung cancer cell line. The ‘plus’ and ‘minus’ symbols indicate whether the extracts have been subjected to heat inactivation (HI). ‘pd’ represents population doublings. (**D**) A Western blot probed with anti-SV40 T-Ag antibodies shows stable expressed of T-Ag only in MRC-5^TSR^ cell. HDAC1 is a loading control. (**E**) MRC-5^TSR^ cells show elevated levels of H-RAS protein due to expression of exogenous H-RAS^V12G^ (see also Supplementary Figure S1D). α-Tubulin is a loading control.

To follow the epigenetic changes that accompany the immortalization and transformation of normal diploid human somatic cells, we generated two isogenic cell lines with defined characteristics (Figure 1A). First, we introduced by retroviral infection the catalytic component of human telomerase (hTERT) into foetal lung fibroblast cell line MRC-5, which normally has a limited life span in culture and enters senescence after 20–25 pd. This step ensured that the immortalized cells stably maintain their telomeres and do not become aneuploid after prolonged culturing (Supplementary Figure S1A and B). After selection for cells expressing hTERT, we sequentially introduced by retroviral infection into a subset of hTERT-expressing cells two oncogenes: the Simian Virus 40 large T-Ag and constitutively active oncogenic H-RAS^G12V^ (Figure 1A). We will refer to these two cell lines as MRC-5^hTERT^ and MRC-5^TSR^, respectively.

Stable expression of hTERT in MRC-5^hTERT^ cells was sufficient to bypass senescence and extend the proliferative life span of the parental MRC-5 cell line beyond one year in culture (Figure 1B). Therefore, the MRC-5^hTERT^ cells can be designated as immortalized, as has been previously reported (13,27). To confirm that the extended life span in culture of MRC-5^hTERT^ cells was due to persistent telomerase activity, we performed a Telomere repeat amplification protocol (TRAP) assays on cell extracts from early (50 pd; 3 months in culture) and late (100 pd; 6 months in culture) passage cells. In parallel, we also examined the MRC-5^TSR^ cells to ask whether telomerase activity is stably maintained in the presence of oncogenes. We detected a characteristic 6-bp laddering in MRC-5^hTERT^ and MRC-5^TSR^ cells at 50 and 100 pd, but not in the parental cell line, and this activity was lost upon heat inactivation of the extracts (Figure 1C). The absence of telomerase activity in MRC-5 cell line is consistent with the limited life span of these cells in culture (Figure 1B). The level of telomerase activity detected in MRC-5^hTERT^ and MRC-5^TSR^ cells was sufficient to extend the average length of telomeres from ∼6 to > 10 kb as indicated by the progressive increase in telomere restriction fragment length (Supplementary Figure S1C).

To confirm that the introduced oncogenes are expressed continuously in MRC-5^TSR^ cells, we performed western blots on extracts from the parental cell line as well as MRC-5^hTERT^ and MRC-5^TSR^ cells at 50 and 100 pd. These experiments detected robust expression of SV40 T-Ag and elevated levels of H-RAS in the MRC-5^TSR^ cell line (Figure 1D and E). Because the H-RAS antibody cannot discriminate between the endogenous and the mutant RAS protein, we also cloned and sequenced cDNA from MRC-5^TSR^ cells. This confirmed the presence of mutant *H-RAS^G^^12^^V^* in MRC-5^TSR^ cell line and detected a 1:14 ratio of wild-type to mutant *H-RAS* messenger RNA (mRNA) (Supplementary Figure S1D). Taken together, these experiments demonstrate that hTERT and the introduced oncogenes, SV40 T-Ag and H-RAS^G12V^, are stably expressed in the transduced MRC-5 cells and that this expression does not change significantly over long periods of time in culture.

### Characterization of immortalized and transformed cell lines

**Figure 2.**
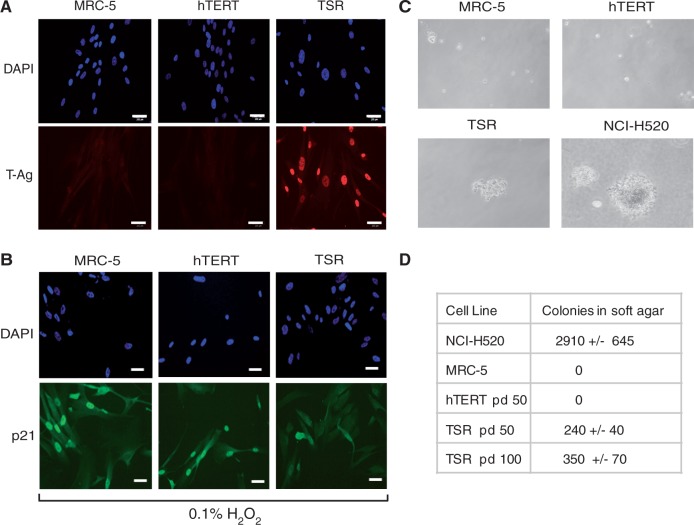
Transformation-induced properties of MRC-5^TSR^ cells (**A**) Immunostaining of MRC-5, MRC-5^hTERT^ and MRC-5^TSR^ cells with antibodies against SV40 T-Ag (red). The cells were counterstained with DAPI (blue). (**B**) Immunostaining of H_2_O_2_-treated MRC-5, MRC-5^hTERT^ and MRC-5^TSR^ cells with anti-p21 antibodies (green) and DAPI (blue). MRC-5^TSR^ cells do not show high levels of p21 induction upon oxidative stress. The scale bars in (A) and (B) represent 200 µm. (**C**) Micrographs of cells grown in soft agar for 4 weeks. Only the MRC-5^TSR^ cells and the control cell line NCI-H520 form colonies in soft agar. (**D**) A table showing the number of colonies formed by MRC-5, MRC-5^hTERT^, MRC-5^TSR^ and NCI-H520 cells in soft agar. The MRC-5^TSR^ cell line was scored at 50 and 100 pd.

The SV40 large T-Ag is known to bind both p53 and pRB tumour suppressor proteins and impair their normal function in controlling cell cycle checkpoints upon cellular stress and cell cycle progression, respectively (28). To determine whether MRC-5^TSR^ cells display reduced p53 and Rb activity and thus can be considered transformed, we investigated their response to oxidative DNA damage and acquisition of anchorage-independent growth. Upon treatment with hydrogen peroxide (H_2_O_2_), we observed accumulation of p53-regulated Cyclin-dependent kinase (CDK) inhibitor protein p21 in the nuclei of MRC-5 and MRC-5^hTERT^ cells, which lack SV40 T-Ag and are expected to have normal p53-dependent response (Figure 2A and B; left and middle panels). In contrast, MRC-5^TSR^ cells expressing SV40 T-Ag did not accumulate nuclear p21 in response to peroxide treatment (Figure 2A and B; right panel), indicating that p53-dependent response to DNA damage is abrogated in these cells.

To examine the acquisition of anchorage-independent growth by the transformed MRC-5 cells, which should be largely dependent on constitutive expression of oncogenic H-RAS (13,29), we scored the ability of MRC-5, MRC-5^hTERT^, MRC-5^TSR^ cells and a control squamous lung carcinoma NCI H-520 cell line to form colonies in soft agar. Consistent with the stable expression of H-RAS^G12V^, MRC-5^TSR^ cells grown for either 50 or 100 pd produced robust colonies in soft agar although with lower frequency than the control H-520 cells (Figure 2C and D). Neither the MRC-5 cells nor the immortalized MRC-5^hTERT^ cell line formed colonies in soft agar (Figure 2C and D). From these experiments, we conclude that MRC-5^hTERT^ cells, although immortal, do not have the characteristic transformed properties of MRC-5^TSR^ cells.

### Progressive accumulation of DNA methylation at gene promoters in immortalized and transformed cell lines

**Figure 3.**
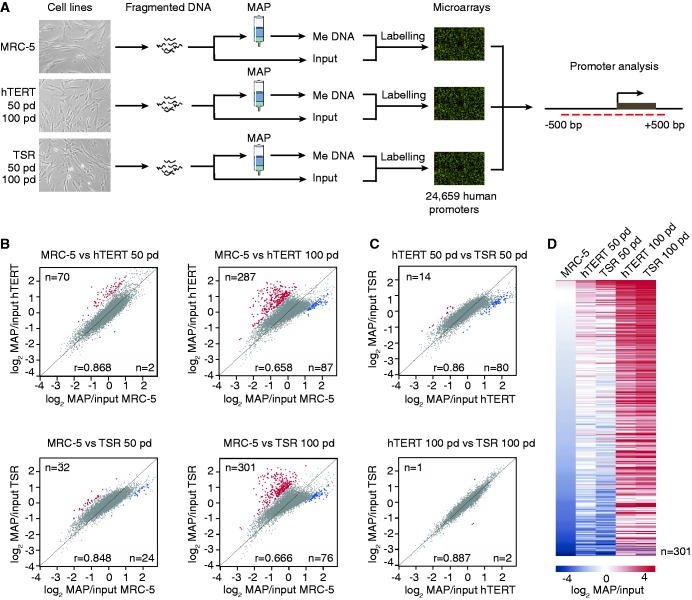
Accumulation of DNA methylation at gene promoters in the immortalized and transformed cell lines. (**A**) Detection of methylated gene promoters in MRC-5 cells, early (50 pd) and late (100 pd) passage MRC-5^hTERT^ and MRC-5^TSR^ cell lines by Methylated DNA Affinity Purification (MAP) coupled with hybridization to promoter microarrays representing 24 659 human RefSeq genes. Regions spanning probes from −500 bp to +500 bp relative to TSS were interrogated. (**B**) Log_2_ plots show differentially methylated gene promoters in early and late passage MRC-5^hTERT^ and MRC-5^TSR^ cells relative to the parental cell line. Promoters displaying ≥2-fold gain of DNA methylation are marked in red. Promoters with ≥2-fold loss of DNA methylation are marked in blue. (**C**) Log_2_ plots comparing DNA methylation patterns at gene promoters between MRC-5^hTERT^ and MRC-5^TSR^ cell lines at early (50 pd) and late (100 pd) passages. (**D**). A heat map visualization of *de novo* methylated gene promoters (*n* = 301) in MRC-5^hTERT^ and MRC-5^TSR^ cell lines at early (50 pd) and late (100 pd) passages in comparison with the parental cell line MRC-5. Promoters methylated at early passage in MRC-5^hTERT^ and MRC-5^TSR^ cells remain methylated in late passage cells.

**Figure 4.**
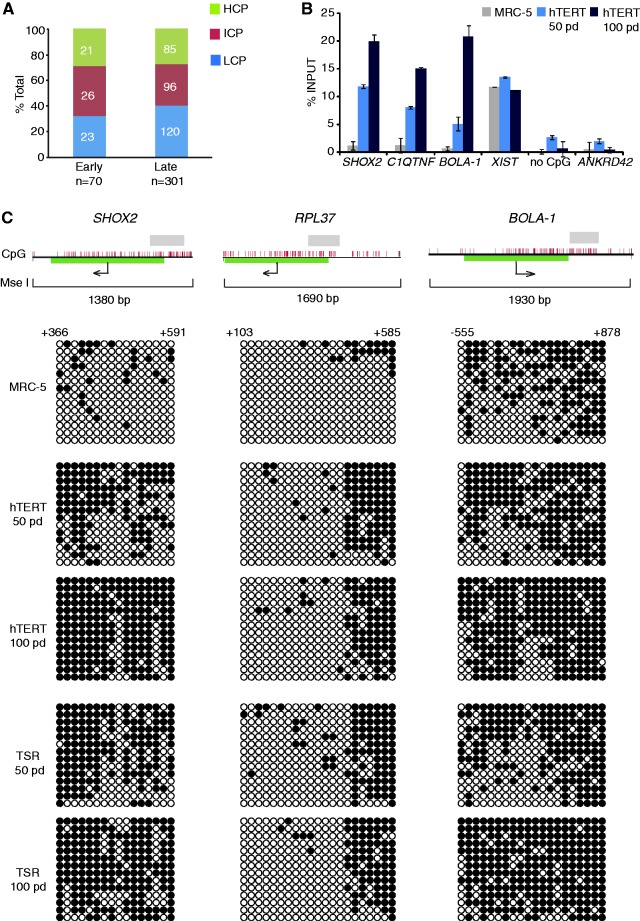
Validation of DNA methylation data obtained from promoter microarray analyses. (**A**) A bar graph representation of low (LCP), intermediate (ICP) and high CpG density (HCP) promoters among loci that are methylated either early or late in MRC-5^hTERT^ cell line. (**B**) DNA methylation levels at early (*SHOX2* and *C1QTNF*) and late (*BOLA-1*) methylating gene promoters in MRC-5 and MRC-5^hTERT^ cell lines detected by MeDIP. Constitutively methylated promoter of the non-coding RNA *XIST* serves as a positive control. *ANKRD42* is a promoter that lacks methylation in all cell lines at any time point. ‘no CpG’ is a region on chromosome X that lacks CpGs. (**C**) Validation of *de novo* DNA methylation at *SHOX2*, *RPL37* and *BOLA-1* gene promoters in MRC-5^hTERT^ and MRC-5^TSR^ cells at 50 and 100 pd by bisulphite DNA sequencing. Methylated CpGs are shown as black circles, unmethylated CpGs as white circles. The graphs at the top of the panel show CpG dinucleotides, 1 kb promoter region analysed by microarray data processing, the span of the MseI restriction fragment and the region analysed by bisulphite DNA sequencing.

We next asked whether DNA methylation patterns remain stable in MRC-5^hTERT^ and MRC-5^TSR^ cells over time and whether the transformation by oncogenes is required to induce changes in DNA methylation at gene promoters that are characteristic of many human tumours. To do so, we used methyl-CpG binding domain affinity purification (MAP) of methylated DNA combined with hybridization to microarrays containing probes for 24 659 human protein-coding RefSeq gene promoters (Figure 3A). To distinguish significant changes in DNA methylation close to TSS from more distal DNA methylation patterns, we analysed the microarray data separately for 1-kb promoter regions (±500 bp from TSS) and the upstream regions (−500 to −1500 bp from TSS), as described previously (17). A minimal cut-off for median log_2_ MAP/input difference between cell lines of 1 (corresponding to 2-fold change in DNA methylation) was used in all analyses. We examined DNA methylation at gene promoters in MRC-5^hTERT^ and MRC-5^TSR^ cells at 50 and 100 pd in culture and compared these values with those for the parental MRC-5 cell line (Figure 3B and Supplementary Table S1). These analyses detected a progressive gain of DNA methylation at gene promoters in immortalized and transformed cells at 50 and 100 pd compared with the parental cell line. However, we detected no significant differences between MRC-5^hTERT^ and MRC-5^TSR^ at either 50 or 100 pd when DNA methylation patterns in these cell lines were compared with each other (Figure 3C). Most *de novo* DNA methylation events (∼250 promoters) occurred late, between 50 and 100 pd, rather than early (32–70 promoters) (Figure 3B and D) and affected promoters with low, intermediate and high CpG density (Figure 4A). Importantly, promoters methylated early (by 50 pd) remained methylated at late passages (100 pd) suggesting that once DNA methylation was established at gene promoters it was stably maintained through subsequent cell divisions (Figure 3D). *De novo* DNA methylation events did not affect preferentially genes located close to telomeres but occurred at loci distributed throughout the genome (Supplementary Figure S2). This suggests that gain of DNA methylation at gene promoters in MRC-5^hTERT^ and MRC-5^TSR^ cells did not result from spreading of subtelomeric heterochromatin from the extended telomeres.

We further confirmed the presence and timing of DNA methylation at gene promoters by MeDIP (24), followed by quantitative PCR at specific early (*SHOX2* and *C1QTNF*) and late (*BOLA-1*) methylated promoters (Figure 4B), as well as by bisulphite DNA sequencing of three selected promoters (*SHOX2*, *RPL37* and *BOLA-1*) in the parental cell line as well as in the MRC-5^hTERT^ and MRC-5^TSR^ cells at 50 and 100 pd (Figure 4C). In all cases, the MeDIP assays and bisulphite DNA sequencing were in agreement with the microarray data.

Taken together, these analyses indicate that identical time-dependent changes in DNA methylation at gene promoters occur in two independent cell populations and that gain of DNA methylation at promoters does not require the presence of oncogenes, such as SV40 T-Ag and oncogenic H-RAS.

### *De novo* DNA methylation occurs predominantly at inactive gene promoters

It has been reported that promoters that carry chromatin marked by Polycomb Repressive Complex 2 (PRC2)-dependent repressive histone H3 lysine 27 trimethylation (H3K27me3) are more susceptible to *de novo* DNA methylation during differentiation of embryonic stem cells to neurons than active promoters marked by H3K4me3 (30). A further correlation between H3K27me3 at gene promoters in non-transformed cells and gain of DNA methylation at such sites in lung, colorectal and breast cancer cell lines has been observed in several independent studies (14–16). This led to the suggestion that in tumours there is a frequent switch from the potentially reversible Polycomb-mediated gene silencing to a more stable long-term repression by DNA methylation (15).

**Figure 5.**
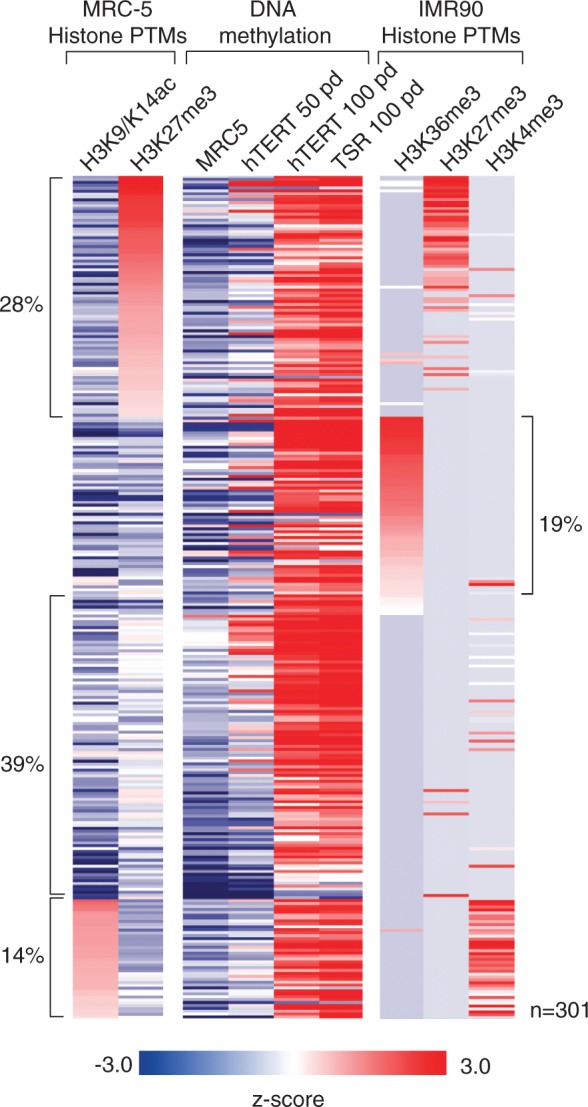
Histone modifications at gene promoters that undergo *de novo* DNA methylation in the immortalized cells. A heat map representation of post-translational histone modifications (PTMs) found in the parental MRC-5 cell line and a related foetal lung fibroblast cell line IMR90 at gene promoters that become methylated in MRC-5^hTERT^ and MRC-5^TSR^ cell lines. Antibodies against histone H3 acetylated at K9 and K14 or trimethylated at K27 were used for ChIP coupled with hybridization to promoter microarrays. Publicly available data for histone PTMs for IMR90 cells was used in these analyses. Only H3K36me3, H3K27me3 and H3K4me3 data for IMR90 cells are shown. Except these three modifications and H3/H4 acetylation (not shown), no other modifications were found significantly enriched at this set of promoters.

To examine whether *de novo* DNA methylation in MRC-5^hTERT^ and MRC-5^TSR^ cells occurs preferentially at promoters that are either pre-marked by H3K27me3 or at those associated with actively transcribed genes, we analysed H3K27me3 and H3 acetylation at gene promoters in the parental MRC-5 cells by ChIP combined with hybridization to promoter microarrays, as described earlier in the text. These experiments showed that 28% of all promoters that acquire DNA methylation in MRC-5^hTERT^ and MRC-5^TSR^ cells carry H3K27me3 in MRC-5 cells and only 14% are enriched in H3 acetylated chromatin (Figure 5). Both active promoters, enriched in acetylated H3, and Polycomb-silenced loci, enriched in H3K27me3 (with few exceptions), displayed a tendency to be methylated late, by 100, but not by 50 pd, suggesting that both modifications delay the appearance of DNA methylation. However, 58% of promoters that become *de novo* methylated in MRC-5^hTERT^ and MRC-5^TSR^ cells had neither H3K27me3 nor acetylated H3 in the parental cell line.

Multiple histone modifications have been mapped by high-throughput approaches in IMR90 fibroblast cell line, which similar to MRC-5, is derived from human foetal lung. Comparison between the two cell lines revealed broadly similar patterns of H3K27me3 and histone acetylation at gene promoters as well as presence of H3K4me3 at loci carrying acetylated H3 (Figure 5). Given the similarity of chromatin modification between IMR90 and MRC-5 cells, we sought to determine whether other histone modifications present at loci that lack either H3K27me3 or H3 acetylation in MRC-5 cells could potentiate gain of DNA methylation at gene promoters in MRC-5^hTERT^ and MRC-5^TSR^ cells. Of all the 22 histone modifications examined in the IMR90 cells, only H3K36me3, normally present within transcribed regions of the genome (31–33), was apparent at 19% of gene promoters that acquire DNA methylation in MRC-5^hTERT^ and MRC-5^TSR^ cells (Figure 5). Interestingly, many of the H3K36me3-marked promoters represent alternative downstream TSSs, which drive the expression of truncated variant transcripts (Supplementary Figure S3). Importantly, about half of the loci that were methylated early (by 50 pd) in the MRC-5^hTERT^ and MRC-5^TSR^ cells showed enrichment for H3K36me3 in the primary parental cell line (Figure 5). Taken together, these analyses indicate that promoters of silenced genes that are either devoid of known modifications or enriched for H3K36me3, a modification refractive to initiation of transcription (31), are prone to DNA methylation early in immortalized human cells, whereas promoters of either actively transcribed or Polycomb-silenced genes tend to be methylated late, by 100 pd. However, none of the examined chromatin modifications can be considered predictive of whether or not a gene promoter will become *de novo* methylated in immortalized cells. Many loci carrying similar histone marks did not accumulate DNA methylation in MRC-5^hTERT^ and MRC-5^TSR^ cells at late passage in culture.

### Immortalized and transformed cells progressively accumulate changes in gene expression

**Figure 6.**
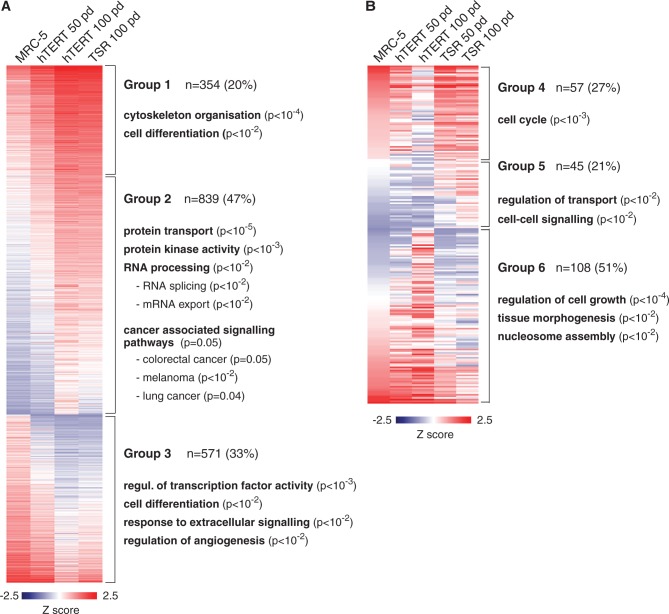
Changes in gene expression in immortalized and transformed cell lines. (**A**) A heat map showing immortality-associated changes in gene expression in MRC-5^hTERT^ and MRC-5^TSR^ cell lines at 50 and 100 pd. Three groups of genes can be clearly distinguished. The most significant functions of representative up- and downregulated groups of genes identified by gene ontology and gene set enrichment analyses are indicated. (**B**) A heat map representation of transformation-associated changes in gene expression in MRC-5^TSR^ cell line in comparison with the primary MRC-5 and immortalized MRC-5^hTERT^ cells. The most significantly enriched biological functions attributed to the three groups of genes are indicated.

Given that in MRC-5^hTERT^ cells, we observed gain of DNA methylation primarily at promoters of genes that were inactive in the parental cell line, we asked whether gene expression patterns in hTERT-immortalized cells remain stable after 50 and 100 pd in culture. We also sought to determine whether expression of SV40 T-Ag and constitutively active H-RAS^G12V^ in MRC-5^TSR^ cells had significant role in reprogramming gene expression profiles as reported for short-term studies of human cells transformed by viral oncogenes (34,35). To address these questions, we used microarrays to examine gene expression patterns in MRC-5^hTERT^ and MRC-5^TSR^ cells at 50 and 100 pd and compared these to each other and to the parental MRC-5 cell line. Surprisingly, we found that cells immortalized by hTERT progressively accumulate significant changes in gene expression, which were also shared by the MRC-5^TSR^ cells (Figure 6A). Thus, we detected 1193 transcripts that were upregulated and 571 transcripts that were downregulated by 3-fold or more in immortalized and transformed cells by 100 pd compared with the parental cell line (Supplementary Table S2). Upregulated transcripts could be divided into two distinct groups: genes that were weakly expressed in the MRC-5 cells, but upregulated in MRC-5^hTERT^ and MRC-5^TSR^ cell lines (Group 1; upregulated), and genes that were expressed in MRC-5^hTERT^ and MRC-5^TSR^ cells, but not in the parental cell line (Group 2; activated) (Figure 6A). Gene ontology and gene set enrichment analyses showed that transcripts from Group 1 included proteins involved in cytoskeletal organization and cell migration, whereas many of the transcripts from Group 2 encode for proteins implicated in cancer-associated signalling pathways, regulation of MAP kinase cascade, protein transport and RNA splicing (Figure 6A). The transcripts downregulated in MRC-5^hTERT^ and MRC-5^TSR^ cells (Group 3) were enriched in regulators of cell differentiation, modulation of transcription factor activity and proteins involved in response to extracellular signalling. Interestingly, a number of genes that change their expression in immortalized cells, e.g. *PI3K*, *MDM2*, *SMAD2/3* and *STAT1* (Supplementary Figure S4), are implicated in the evasion of apoptosis and acquisition of insensitivity to growth-inhibiting signals, which are characteristic features of tumour cells. We validated these expression changes by independently performed quantitative reverse transcription PCRs on several selected transcripts (Supplementary Figure S6A and B).

We detected a much smaller number of up- and downregulated transcripts (210) that could be attributed to the constitutive expression of oncogenes, as the levels of these mRNAs were different between MRC-5^hTERT^ and MRC-5^TSR^ cells (Figure 6B and Supplementary Table S2). Here, we also identified three distinct groups of transcripts (labelled Groups 4, 5 and 6). The mRNAs from Group 4 were downregulated in late passage MRC-5^hTERT^ cells, but these were highly expressed in MRC-5 as well as MRC-5^TSR^ cells and included genes involved in cell cycle regulation such as *Securin*, *CDC25* phosphatase and the kinase *Aurora B*. The transcripts from Group 5 were enriched for regulators of transport and cell–cell signalling and were expressed neither in MRC-5 nor in MRC-5^hTERT^ cells, but were progressively upregulated exclusively in the transformed MRC-5^TSR^ cell line (Figure 6B). Finally, Group 6 included transcripts that were upregulated in the immortalized MRC-5^hTERT^ cells, but expressed in the MRC-5^TSR^ cell at levels comparable with the parental cell line. This group was enriched in regulators of cell growth, tissue morphogenesis and nucleosome assembly. As expected, many of the proteins with altered levels of expression in MRC-5^TSR^ cells belong to cancer-associated signalling pathways and have roles in promoting cellular proliferation, angiogenesis and cell survival (Supplementary Figure S5). Although some of these components are upregulated already in MRC-5^hTERT^ cells, their levels of expression are further enhanced upon introduction of oncogenes. Independently performed quantitative reverse transcription PCRs on a subset of transcripts were in agreement with the microarray data (Supplemental Figure S6C and D).

Taken together, these analyses demonstrate that sustained expression of hTERT leads to significant and complex large-scale reprogramming of the transcriptional output of the genome, which is likely to reflect adaptation to highly proliferative state. On the other hand, expression of SV40 T-Ag and oncogenic H-RAS^V12G^ in hTERT-immortalized cells induces fewer sustainable changes in gene expression, but these might be essential for tumorigenisity and acquisition anchorage-independent growth.

## DISCUSSION

Aberrant DNA methylation at gene promoters has been reported for many tumours and typically is accompanied by lack of transcription from the associated gene. Although there are many specific examples of silencing of tumour suppressor genes by promoter DNA methylation, recent high-throughput analyses in breast, colorectal and other types of cancer have suggested that the vast majority of gene promoters methylated in tumours represent developmentally regulated loci, which are already repressed in pre-cancerous tissues (36,37). These observations highlight the coexistence of ‘driver’ and ‘passenger’ *de novo* methylation events that occur in tumours implying that most changes in DNA methylation at gene promoters are unlikely to contribute to cancer formation (15,38,39). Nevertheless, several important questions arise from these studies. How are the aberrant patterns of DNA methylation brought into existence? What are the dynamics of *de novo* DNA methylation and the molecular determinants of this process? Are epigenetic alterations linked intrinsically to genetic determinants of tumour formation?

To address some of these questions, we used a model system, which allows defined genetic components to be sequentially introduced into primary human cells with normally finite life in culture. The contribution of these genetic components to changes in growth characteristics of modified cells, gene expression patterns and promoter DNA methylation could then be examined by high-throughput assays. Thus, the expression of the catalytic subunit of telomerase enzyme (hTERT) in MRC-5 foetal lung fibroblasts generated an immortal cell line with life span extended for >200 cell generations, whereas further expression of collaborating oncogenes, SV40 T-Ag and H-RAS^V12G^, in hTERT-immortalized cells produced an isogenic transformed cell line characterized by acquisition of anchorage-independent growth. Our detailed investigation of promoter DNA methylation in these two isogenic cell lines identified loci that are prone to time-dependent *de novo* DNA methylation and led us to conclude that the changes in DNA methylation at promoters do not require expression of oncogenes. Near identical changes in DNA methylation at gene promoters took place in the immortalized (MRC-5^hTERT^) and transformed (MRC-5^TSR^) cell lines with stable diploid karyotype. This is somewhat surprising given that constitutively active K-RAS and H-RAS have been implicated in DNA methylation-mediated silencing of specific genes (40,41). In contrast to these findings, our data firmly suggest that cellular immortality conferred by hTERT expression is sufficient to promote *de novo* DNA methylation at gene promoters. Whether the immortal and transformed cells display differences in DNA methylation elsewhere in the genome is yet to be determined.

In agreement with recent studies (42), the vast majority of *de novo* DNA methylation events in MRC-5^hTERT^ and MRC-5^TSR^ cell lines occurred at promoters of genes that were already silenced in the parental cell line. Some of these represent loci carrying repressive H3K27me3 and H3K36me3 histone modifications. However, it seems unlikely that histone modifications determine whether or not a promoter will become methylated in immortalized cells. About 40% of the loci hypermethylated in MRC-5^hTERT^ and MRC-5^TSR^ cells were devoid of H3K27me3 and H3K36me3 in the parental cell line and had no other detectable known modifications in the closely related IMR90 fibroblasts. Taken together, these data suggest that lack of promoter activity and, potentially, stably bound transcription factors, which could protect such loci against DNA methylation machinery (43,44), may result in gradual acquisition of DNA methylation over time. Our data also indicate that promoters of actively transcribed genes, marked by H3 acetylation and H3K4me3, tend to be more stably protected. Few active promoters became methylated in the immortal cells, and in all cases this occurred at late passage. In contrast to silenced genes, methylation of active promoters could represent rare driver methylation events, which promote cell proliferation and survival. It is plausible that stochastic DNA methylation events take place in immortalized cells, and these patterns are under constant surveillance and selection. Therefore, only those methylation events that occur either at weakly protected silenced promoters or genes, inactivation of which favours long-term survival, will be tolerated and stably propagated in the immortal cell populations. As immortality, and in many cases the expression of hTERT (45), is a hallmark of all tumours, this may explain why aberrant DNA methylation is such a prevalent feature in a variety of cancer cell types.

Another essential feature of hTERT-immortalized cells is time-dependent acquisition of large-scale changes in gene expression (42,46). Given the stable diploid karyotype of MRC-5^hTERT^ cells, these expression patterns must be epigenetic by nature, as they cannot be explained by aneuploidy or alterations in DNA sequence. In contrast to *de novo* DNA methylation events, the changes in gene expression observed in MRC-5^hTERT^ cells are likely to result from selective pressure to enhance traits that favour long-term survival and stable proliferation in culture. As the evasion of apoptosis, effective repair of DNA damage and robust progression through the cell cycle are essential properties of tumour cells, it is probably not surprising that proteins with known function in cancer-associated signalling pathways show altered expression in the immortal cells. Although subsequent introduction of SV40 T-Ag and constitutively active H-RAS into hTERT-immortalized cells results in fewer high-amplitude changes in gene expression, our data indicate that the presence of cooperating oncogenes promotes subtle alterations in many signalling pathways, confers insensitivity to growth signals and acquisition of anchorage-independent growth. Taken together, these observations imply that telomerase-induced immortality is sufficient for large-scale reprogramming of DNA methylation at gene promoters and expression patterns in diploid human cells to a state that resembles pre-cancerous lesions. Such reprogramming reflects the intrinsic plasticity of immortal cell genome which, in combination with oncogene-dependent modulation of responses to stress and growth signals, may favour adaptation to a variety of cellular and tissue microenvironments and ultimately support tumour growth.

## SUPPLEMENTARY DATA

Supplementary Data are available at NAR Online.

## FUNDING

This research was supported by Cancer Research UK Senior Fellowship [C7215/A8983] and EMBO Long-term fellowship (to T.C.). The Wellcome Trust Centre for Cell Biology is supported by core funding from the Wellcome Trust [092076]. Funding for open access charge: The Wellcome Trust via University of Edinburgh.

*Conflict of interest statement*. None declared.

## Supplementary Material

Supplementary Data
