# Supplementary material for: Immortality, but not oncogenic transformation, of primary human cells leads to epigenetic reprogramming of DNA methylation and gene expression
Source: Nucleic Acids Res. 2013 Dec 26;42(6):3529–41. doi: 10.1093/nar/gkt1351 (PMC3973294; doi:10.1093/nar/gkt1351)
Supplement: Supplementary Data [file supp_42_6_3529__index.html]

Immortality, but not oncogenic transformation, of primary human cells leads to epigenetic reprogramming of DNA methylation and gene expression — Immortality, but not oncogenic transformation, of primary human cells leads to epigenetic reprogramming of DNA methylation and gene expression — Supplementary Data 

# Immortality, but not oncogenic transformation, of primary human cells leads to epigenetic reprogramming of DNA methylation and gene expression

## Supplementary Data

files

**Files in this Data Supplement:**

- Supplementary Data - pdf file
- Supplementary Data - xls file
- Supplementary Data - xls file
