# Supplementary material for: Immortality, but not oncogenic transformation, of primary human cells leads to epigenetic reprogramming of DNA methylation and gene expression
Source: Nucleic Acids Res. 2013 Dec 26;42(6):3529–41. doi: 10.1093/nar/gkt1351 (PMC3973294; doi:10.1093/nar/gkt1351)
Supplement: Supplementary Data [file supp_gkt1351_nar-02408-x-2013-File008.pdf]

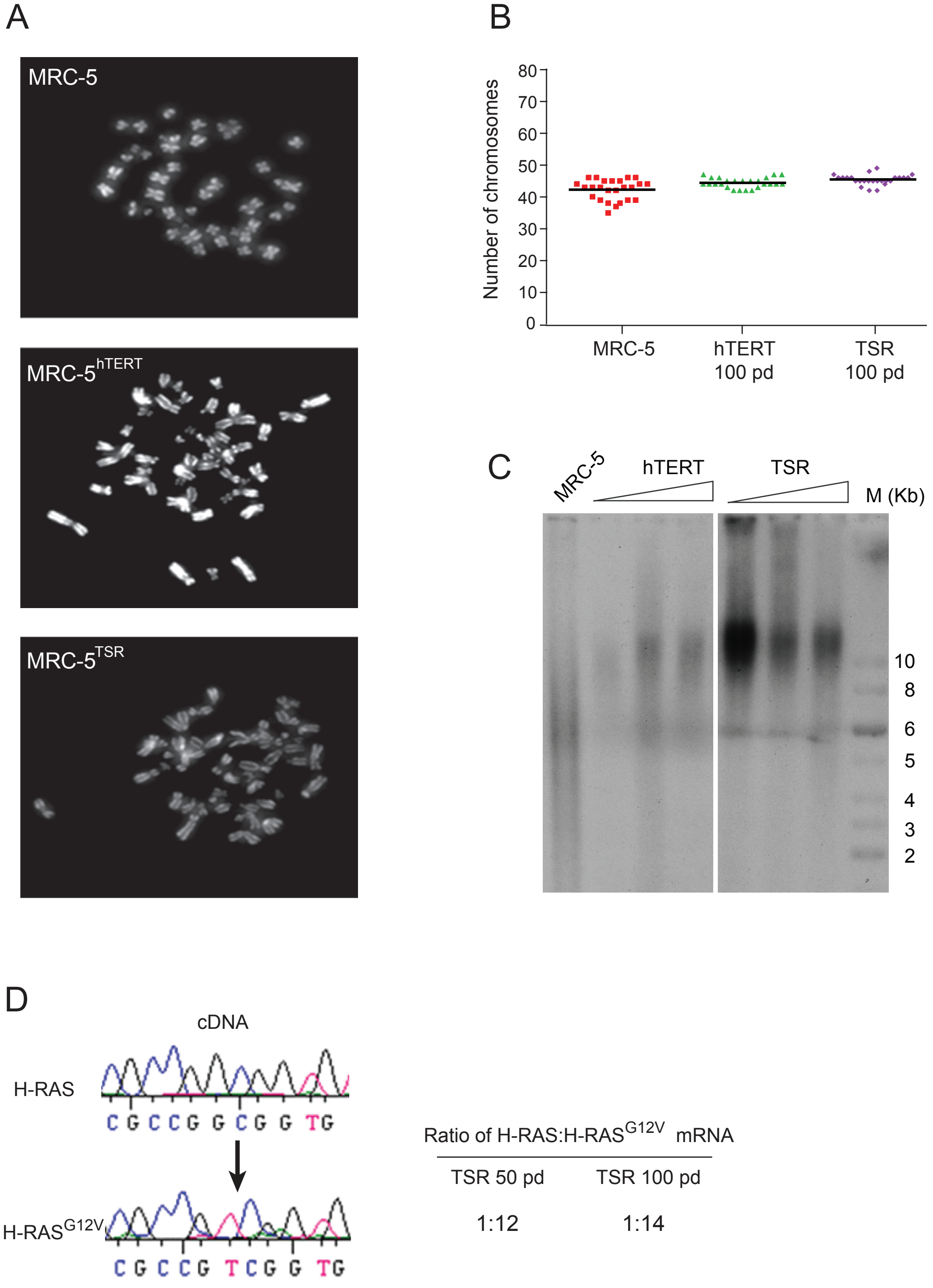

Supplemental Figure 1 Characterization of MRC-5^{hTERT} and MRC-5^{TSR} cell lines

A. Representative metaphase spreads from MRC-5, MRC-5^{hTERT} and MRC-5^{TSR} cell lines. Immortalized and transformed cells were grown for 100 population doublings. **B.** Chromosome counts per metaphase spread for MRC-5, MRC-5^{hTERT} and MRC-5^{TSR} cell lines. The chromosomes from 25 metaphase spreads for each cell line were counted. **C.** The length of telomeres in the parental MRC-5 cell line, MRC-5^{hTERT} and MRC-5^{TSR} cells grown for 50, 75, 100 population doublings was investigated by Southern blot hybridization on HinfI and RsaI digested DNA with γP^{32}-labelled [(TTAGGG)$_3$] probe. Note that the MRC-5 cells have telomeres with heterogeneous size which upon expression of hTERT become stabilized at ~10 Kb size. **D.** Quantification of wild-type and mutant *H-RAS* expression in MRC-5^{TSR} cells at 50 and 100 population doublings. PCR products, obtained from cDNA amplification with primers flanking the point mutation, were cloned and sequenced. The calculated ratio of wild type *H-RAS* to mutant *H-RAS*^{G12V} mRNA at 50 pd and 100 pd was 1:12 and 1:14, respectively .

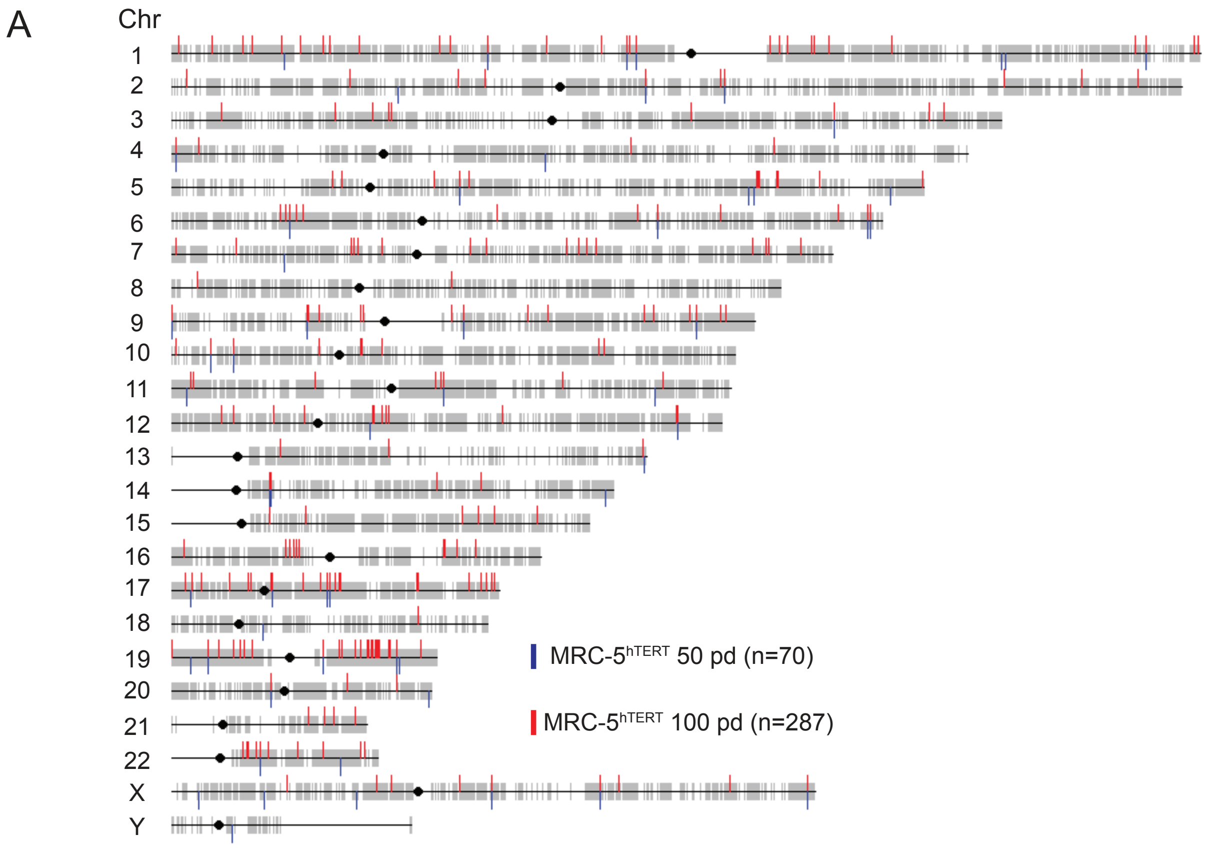

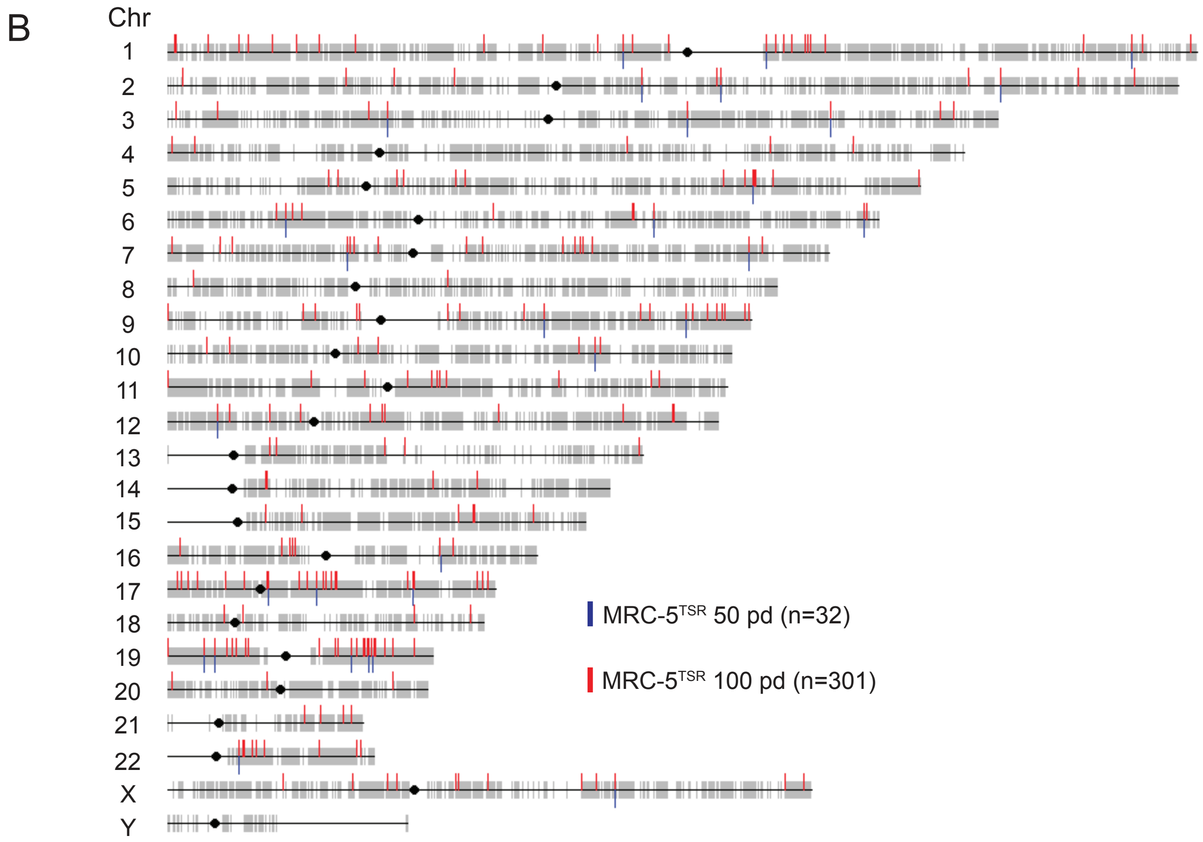

Supplemental Figure 2 Chromosomal maps of *de novo* methylated gene promoters in MRC-5^{hTERT} and MRC-5^{TSR} cells

A. Distribution of *de novo* methylated gene promoters across the genome in MRC-5^{hTERT} cells at 50 (blue) and 100 (red) population doublings (pd). The gray bars indicate all annotated RefSeq promoters.
B. Distribution of *de novo* methylated gene promoters across the genome in MRC-5^{TSR} cells at 50 (blue) and 100 (red) population doublings. Note that *de novo* DNA methylation does not occur preferentially in subtelomeric regions.

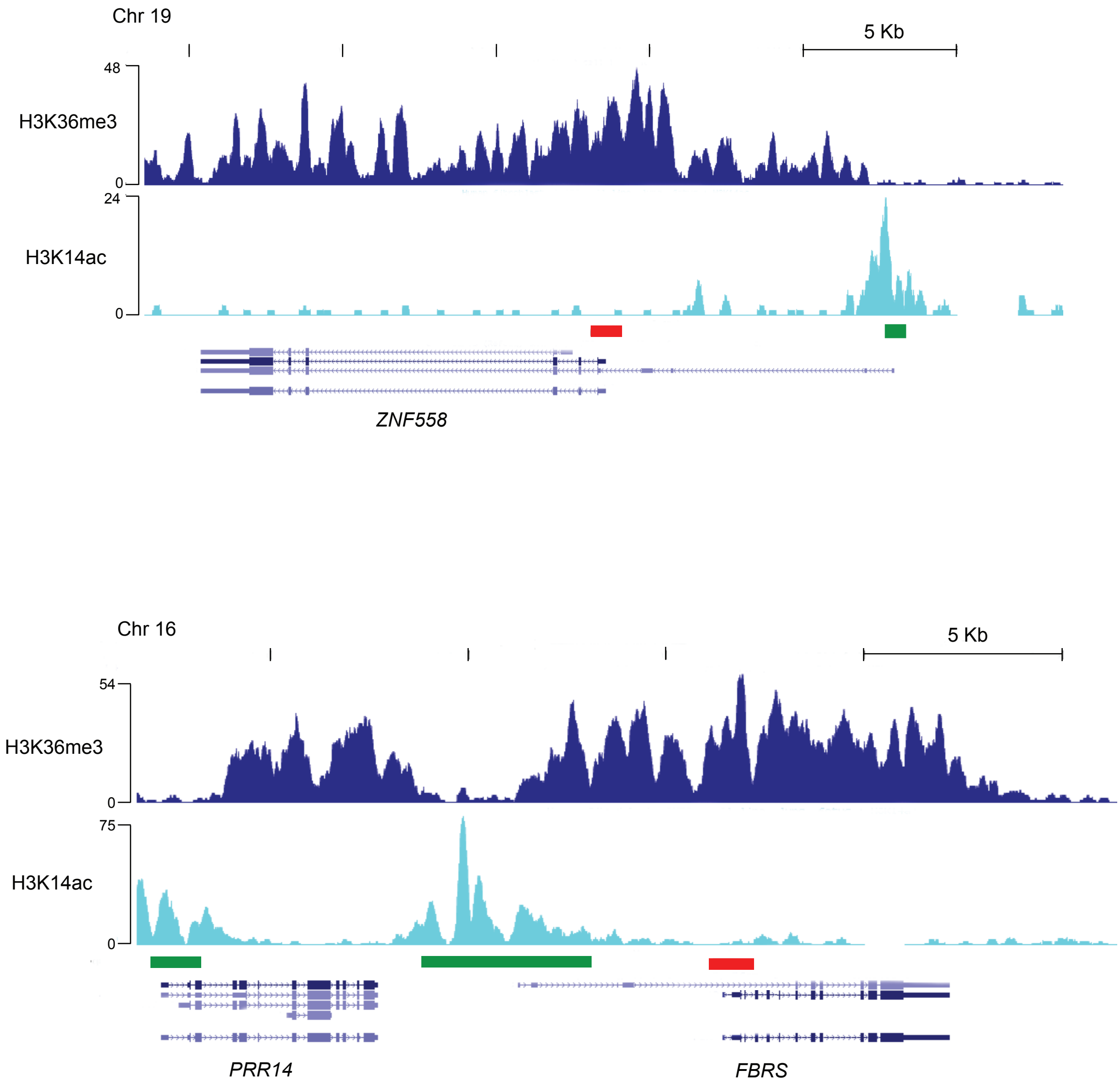

Supplemental Figure 3 Examples of alternative transcription start sites marked by H3K36me3 in IMR90 cells

Images generated by UCSC genome browser from data produced by the Roadmap Epigenomics project showing H3K36me3 and H3K14 acetylation at *ZNF558* and *FBRS* loci located on chromosomes 19 and 16, respectively. The green boxes represent CpG islands. The red boxes indicate CpG islands that coincide with alternative transcription start sites (TSS) and carry transcription elongation-dependent H3K36me3. These alternative TSS undergo time-dependent *de novo* DNA methylation in the immortalized MRC-5 cells.

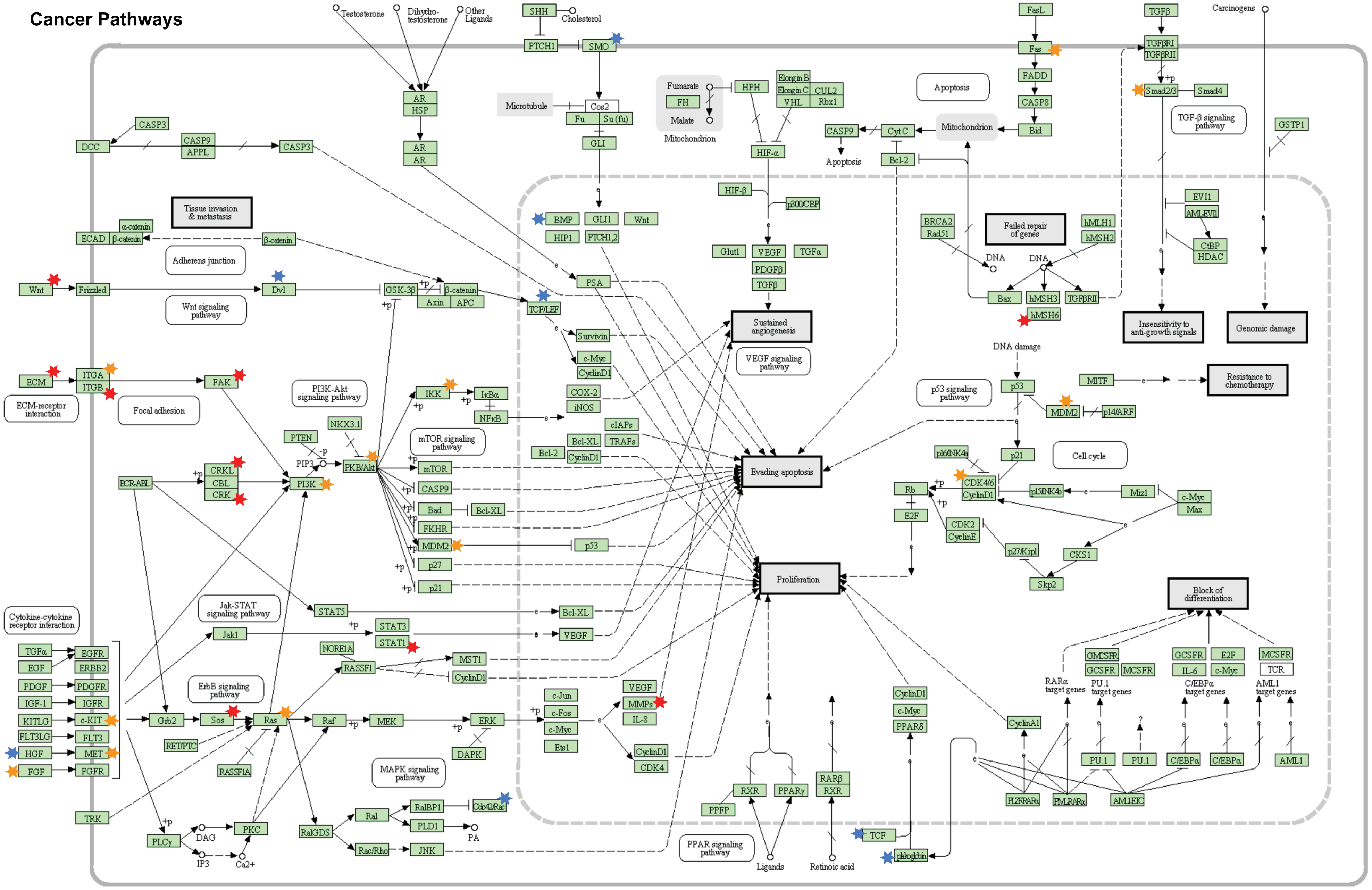

Supplemental Figure 4 Components of cancer-associated pathways misregulated in hTERT-immortalised cells

A diagram generated by KEGG pathway maps (www.genome.jp/kegg) indicates specific genes that are up-regulated (red; Group I), activated (orange; Group II) or repressed (blue; Group III) by 3-fold or more in MRC-5^{hTERT} and MRC-5^{TSR} cell lines.

Cancer Pathways

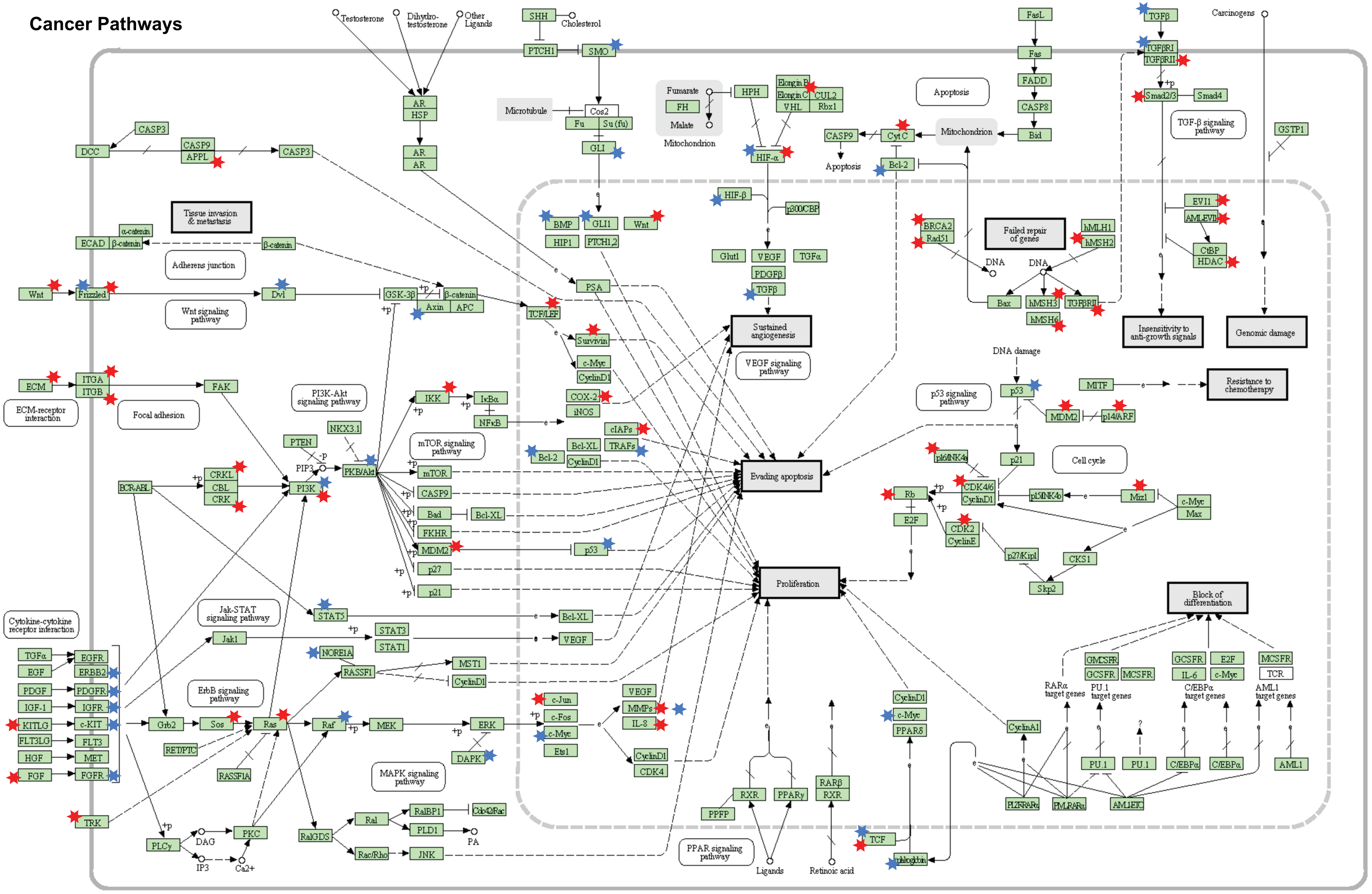

Supplemental Figure 5 Transformation-associated changes of gene expression affect multiple pathways

A KEGG cancer pathways diagram shows genes that are either upregulated (red) or downregulated (blue) by 2-fold or more in the transformed MRC-5^{TSR} cell line relative to the immortalised MRC-5^{hTERT} cell line at 100 pd.

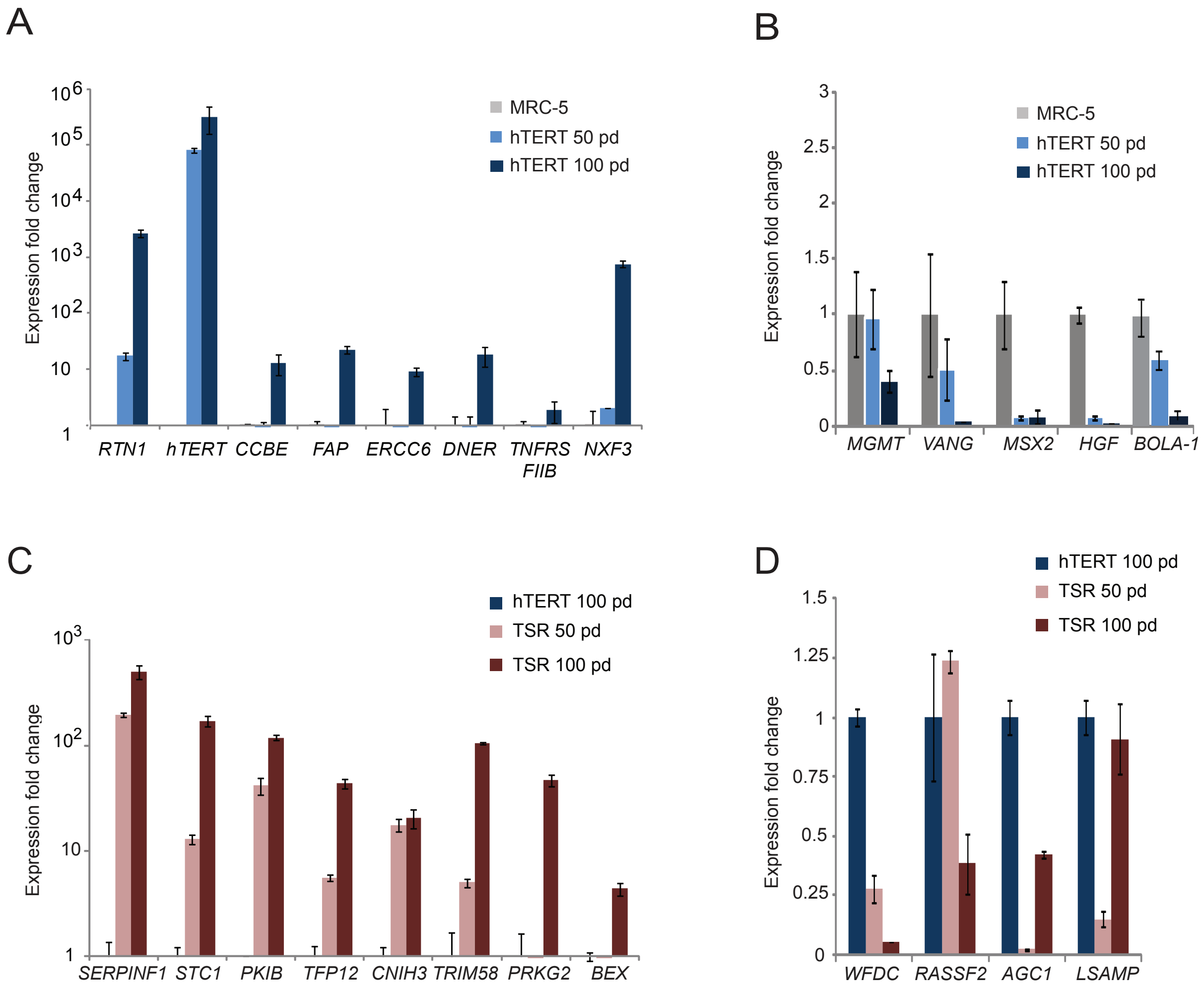

Supplemental Figure 6   Validation of changes in gene expression by quantitative RT-PCR

A-B. Transcripts up- and downregulated in hTERT-immortalised MRC-5 cells.
C-D. Transcripts up- and downregulated in transformed, but not in hTERT-immortalised cells.
